# Andrographolide Ameliorates Inflammatory Changes Induced by D-Lactate in Bovine Fibroblast-like Synoviocytes

**DOI:** 10.3390/ani14060936

**Published:** 2024-03-19

**Authors:** Stefanie Teuber, Carolina Manosalva, Pablo Alarcón, John Quiroga, Diana Pantoja, María Angélica Hidalgo, Gabriel Morán, Rafael Agustín Burgos

**Affiliations:** 1 Laboratory of Inflammation Pharmacology and Immunometabolism, Institute of Pharmacology and Morphophysiology, Faculty of Veterinary Sciences, Universidad Austral de Chile, Valdivia 5090000, Chile; stefanie.teuber@uach.cl (S.T.); pabloalarcon.u@gmail.com (P.A.); john.quiroga@uach.cl (J.Q.); diana.pantoja@uach.cl (D.P.); mahidalgo@uach.cl (M.A.H.); 2 Institute of Pharmacy, Faculty of Sciences, Universidad Austral de Chile, Valdivia 5090000, Chile; carolinamanosalva@uach.cl

**Keywords:** bovine fibroblast-like synoviocytes, D-lactate, TNFα, andrographolide, inflammation

## Abstract

**Simple Summary:**

In cattle experiencing acute ruminal acidosis, joint inflammation causing lameness has been associated with a molecule called D-lactate. We investigated whether the natural compound, andrographolide, could alleviate the inflammation and associated metabolic changes in the joint cells, known as fibroblast-like synoviocytes. We conducted tests by culturing these cells in the presence or absence of andrographolide and observed its impact on the expression of certain markers related to inflammation and metabolism. Our findings revealed that andrographolide reduced the levels of inflammatory markers and metabolic changes triggered by D-lactate. Essentially, it displayed the potential for reversing these harmful effects in the joint cells of cattle. These discoveries hold promise for potential treatments to manage joint inflammation and related issues in cattle, which could significantly benefit their health and welfare.

**Abstract:**

During acute ruminal acidosis, the manifestation of aseptic polysynovitis and lameness in cattle has been observed. Evidence suggests that joint inflammation can be attributed to the metabolic alterations induced by D-lactate in fibroblast-like synoviocytes (FLSs). We aimed to investigate whether andrographolide could mitigate the inflammation and metabolic alterations induced by D-lactate in bovine fibroblast-like synoviocytes (bFLSs). To assess this, bFLSs were cultured in the presence or absence of andrographolide. We evaluated its potential interference with the expression of proinflammatory cytokines, COX-2, HIF-1α, and LDHA using RT-qPCR. Furthermore, we investigated its potential interference with PI3K/Akt signaling and IκBα degradation through immunoblotting and flow cytometry, respectively. Our observations revealed that andrographolide reduced the elevation of IL-6, IL-8, COX-2, HIF-1α, and LDHA induced by D-lactate. Additionally, andrographolide demonstrated interference with the PI3K/Akt and NF-κB pathways in bFLSs. In conclusion, our findings suggest that andrographolide can potentially reverse the inflammatory effects and metabolic changes induced by D-lactate in bFLSs, showing promise as a therapeutic intervention for managing these conditions associated with lameness.

## 1. Introduction

Lactate, once considered solely a metabolic waste product, is now recognized as a pleiotropic signal involved in various physiological and pathological conditions [1,2,3,4]. Lactate exists in two enantiomers: L-lactate, which is produced during glycolysis under anaerobic conditions, and D-lactate, which is generated through the methylglyoxal detoxification pathway [1].

During ruminal acidosis in cattle, an excessive consumption of highly digestible carbohydrates leads to a rumen fermentative disorder, resulting in an increased production of L/D-lactate [5]. However, L-lactate is easily metabolized by cytosolic lactate dehydrogenase (LDH), while D-lactate tends to accumulate from lactate-producing bacteria in the rumen and has a slower metabolic rate in some tissues [5,6,7,8]. The accumulation of D-lactate in the rumen and plasma can contribute to the metabolic disturbances observed during ruminal acidosis [5,9].

Ruminal acidosis indeed can lead to a reduced feed intake and milk production, causing digestive problems such as diarrhea, dehydration, and electrolyte imbalances. In severe cases, it can result in conditions like lameness [5,6,10].

In this sense, an increase in the D-lactate levels in synovial fluid, reaching approximately 5 mM, occurs after the experimental induction of ruminal acidosis in dairy heifers [11]. During ruminal acidosis, lameness is observed in animals, which is associated with the presence of laminitis and polysynovitis in cattle [5,10,12]. Inflammatory joint conditions are characterized by the inflammation of the synovial tissue, which involves the activation of fibroblast-like synoviocytes (FLSs) and the subsequent production of inflammatory mediators. We previously reported an increase in polymorphonuclear leukocytes (PMNs) and inflammatory markers, including IL-6, IL-8, and PGE2, in the synovial fluid of animals with an overload of oligofructose through experimentally induced ruminal acidosis [12]. This finding suggested that D-lactate could exert local inflammatory effects in the synovium. Lactate promotes hypoxia-inducible factor 1-alpha (HIF-1α) activation, encompassing metabolic reprogramming and IL-6 activation in fibroblasts [13] and RA-FLS [14,15]. These findings suggest a close relationship between HIF-1α and the expression of IL-6, as well as metabolic rewiring. The modulation of HIF-1α expression has shown correlation with Nuclear Factor-kappa B (NF-κB) activation. Indeed, a pivotal role of the NF-κB pathway in the expression of HIF-1 mRNA has been demonstrated in IL-17A-stimulated RA-FLS [16]. Moreover, lactate has been involved in the activation of the NF-κB pathway induced by TNF-α in rheumatoid arthritis fibroblast-like synoviocytes (RA-FLSs) [17]. In bovine fibroblast-like synoviocytes (bFLSs), 5 mM of D-lactate has been shown to activate the PI3K/Akt/NF-κB pathway, thereby controlling the expression of proinflammatory cytokines directly [18] or through the hypoxia-inducible factor (HIF-1) [19]. Additionally, the activating of the HIF-1 pathway by D-lactate induces metabolic reprogramming, including an increase in the expressions of GLUT-1 and LDH subunit A (LDHA) [19]. This increase in the glycolytic metabolism is a crucial cellular adaptation during inflammatory processes in joint diseases.

Lameness treatment in cattle using Nonsteroidal Anti-Inflammatory Drugs (NSAIDs) poses challenges due to their inconsistent clinical efficacy. Consequently, there is a lack of approved analgesic drugs for lactating dairy cattle affected by lameness [20,21]. Developing new anti-inflammatory molecules with alternative targets is necessary to overcome these limitations and improve management strategies for lameness and associated pain in cattle.

Andrographolide is a labdane diterpene isolated from the herb *Andrographis paniculata*. Some clinical studies suggest that the use of *Andrographis paniculata* standardized to andrographolide improves clinical parameters in patients with osteoarthritis and rheumatoid arthritis, alleviating pain and reducing joint swelling [22,23]. Furthermore, this natural compound has demonstrated potent anti-inflammatory effects due to its inhibition of NF-κB pathways, which reduces the degradation of inhibitor-κB-α (IκB-α) in human monocytic THP-1 cells, microglia, and endothelial cells [24,25,26]. Andrographolide has reduced joint inflammation in gout experimental models and Complete Freund’s Adjuvant (CFA)-induced arthritis [27,28]. In RA-FLSs under hypoxic conditions, andrographolide reduces migration, invasion, and matrix metalloproteinase expression by inhibiting HIF-1α signaling [29]. Considering this background, we aimed to assess whether andrographolide can reduce the inflammation and metabolic markers induced by D-lactate in bFLSs, evaluating its potential feasibility as a promising candidate for use as an anti-inflammatory agent in cattle. Our observations revealed that andrographolide effectively decreased the overexpression of IL-8, IL-6, and COX-2 induced by D-lactate in bFLSs. Additionally, andrographolide disrupted the phosphorylation of Akt, prevented the degradation of IkBα, and reduced the expression of HIF-1α and LDHA in bFLSs.

## 2. Materials and Methods

### 2.1. Cell Culture

The bFLS isolation was executed following the methodology detailed by Manosalva et al., 2020 [18]. Briefly, bFLSs were retrieved from the carpometacarpal joint of five black Friesian multiparous dairy cows weighing between 400–500 kg, obtained from a local slaughterhouse. All procedures adhered to the guidelines stipulated by the Agencia Nacional de Investigación y Desarrollo (ANID) based on Chilean Animal Protection Laws. Ethical approval for the study protocol was obtained from the ethical committee of the Universidad Austral de Chile (416-2021, Valdivia, Chile).

The synovial membrane underwent enzymatic digestion using a 0.2% type IV collagenase (Thermo Fisher Scientific, Waltham, MA, USA) for a duration of 2 h at 37 °C with gentle agitation. The resultant cell suspension was cultured in Dulbecco’s Modified Eagle’s Medium/Nutrient Mixture F12 (DMEM/F12; Gibco, Thermo Fisher Scientific) supplemented with a 10% fetal bovine serum (FBS; Cytiva, Marlborough, MA, USA) and 1x antibiotic/antimycotic within a 75 cm^2^ flask. The cells were maintained at 37 °C with 5% CO_2_, and passages 2–6 were utilized for subsequent experiments. We validated bFLS identity using CD14 (#557742, Becton Dickinson, Franklin Lakes, NJ, USA) and Vimentin (#BM5501F, Acris, Herford, Germany) antibodies following established protocols [18].

### 2.2. Quantitative Real-Time Polymerase Chain Reaction

bFLSs were cultured in 6-well plates and exposed to either the vehicle (dH_2_O), 5 mM of D-Lactate (Santa Cruz Biotechnologies, Santa Cruz, CA, USA) or 100 ng/mL of bTNF-α (Thermo Fisher Scientific) for 6 h at 37 °C in a 5% CO_2_ environment. Prior to stimulation, cells were pre-incubated with either the vehicle (0.1% DMSO) or different concentrations of andrographolide (AP; #365645, Sigma-Aldrich, Saint Louis, MO, USA) for 30 min. Subsequently, the supernatant was collected and stored for cytokine detection.

The total RNA extraction from the bFLSs was performed using an RNA I kit (Omega Bio-Tek Inc., Norcross, GA, USA) following the manufacturer’s protocol. RNA was treated with DNase to eliminate genomic DNA (Thermo Fisher Scientific). Equal quantities of RNA (250 µg) were reverse-transcribed using M-MLV Reverse Transcriptase (Promega, Madison, WI, USA). The real-time polymerase chain reaction (PCR) was conducted using Takyon™ Rox SYBR^®^ master mix (Eurogentec, Seraing, Belgium) in a StepOnePlus™ system (Applied Biosystems, Life Technologies, Foster City, CA, USA). The specific primers used were IL-6 F 5′-TCCTGAAGCAAAAGATCGCA-3′ and R 5′-CCCACTCGTTTGAAGACTGC-3′; IL-8 F 5′-AAACGAGGTCTGCCTAAACCC-3′ and R 5′-TCTTGCTTCTCAGCTCTCTTCAC-3′; COX-2 F 5′-GCATAAGCTGCGCCTTTTCA-3′ and R 5′-CAGGAACATGAGGCGGGTAG-3′; LDHA F 5′-GGCTTTCCCAAAAACCGTGT-3′ and R 5′-CCCATGGCAGCTTAATGGGT-3′; HIF-1α F 5′-GGAGTTGGACCTCTGCGATT-3′ and R 5′-GAGGGGAGAAAAGGCACGTC-3′; and RPS9 F 5′-CGCAAAACCTATGTGACCCC-3′ and R 5′-CCTCCAGACCTCACGTTTGT-3′. PCR conditions included 40 cycles at 95 °C for 30 s, 60 °C for 30 s (annealing), and 72 °C for 30 s (extension) [18]. Expression levels were normalized against the housekeeping gene RPS9 (40S ribosomal protein S9) and quantified by the 2^−ΔΔCt^ method following Livak and Smittgen’s [30] guidelines in StepOne™ v2.3 software (Applied Biosystems, Waltham, MA, USA).

### 2.3. Quantification of IL-6 by Enzyme-Linked Immunoassay (ELISA)

Supernatants collected from the qPCR assay underwent centrifugation at 600× *g* for 6 min and were used to determine IL-6 concentrations employing the IL-6 bovine ELISA Kit (#ESS0029, Thermo Fisher Scientific) following the manufacturer’s protocols. A 96-well plate was coated with capture antibodies and incubated overnight. Subsequently, the wells were blocked for 1 h (using a 4% bovine serum albumin [BSA] and 5% sucrose in phosphate-buffered saline [BSA]), followed by the addition of 100 µL of the sample and incubation for 1–2 h. After two washes, the detection antibody was introduced and incubated for 1 h. Following two additional washes, streptavidin was added, and the mixture underwent an extra incubation of 0.5 h. Lastly, we added a TMB substrate solution followed by an incubation of 20 min in darkness before adding 0.16 M of H_2_SO_4_ to stop the reaction [19]. We conducted all the procedures at room temperature. Samples were analyzed at wavelengths of 450 nm and corrected at 550 nm using a Varioskan Flash Reader (Thermo Fisher Scientific) [19].

### 2.4. Immunoblot

To evaluate Akt phosphorylation, 80% confluent bFLSs were pre-incubated with 5 μM of andrographolide, 50 μM of andrographolide, or a vehicle (0.1% DMSO) for 30 min, followed by stimulation with 5 mM of D-lactate for 5 min or 100 ng/mL of bTNF-α for 15 min at 37 °C under 5% CO_2_. Subsequently, cell lysis was performed using a lysis buffer (50 mM of Tris–HCl pH 7.4, 150 mM of NaCl, 1 mM of EDTA, 20 mM of EGTA, 1.5% Triton X-100, 25 mM of DTT, protease and phosphatase inhibitor cocktails), followed by centrifugation at 18,000× *g* for 20 min at 4 °C. Protein concentration was determined using a Bradford reagent (Bio-Rad, Hercules, CA, USA) [18].

We separated 50 μg of protein from 12% SDS-PAGE gels and subsequently transferred it to nitrocellulose membranes. The membranes were blocked with 5% skim milk in TBS/T (20 mM of Tris–HCl, pH 7.6, 137 mM of NaCl, and 0.05% Tween 20) and incubated overnight with an anti-phosphoAkt Ser 473 antibody (#4060, Cell Signaling Technology, Beverly, MA, USA). For loading control, membranes were stripped and probed with an anti-Akt antibody (#4691, Cell Signaling Technology). Finally, we exposed the membranes to a horseradish peroxidase (HRP)-conjugated anti-rabbit IgG secondary antibody (LI-COR Biosciences, Lincoln, NE, USA), and performed band visualization using the Odyssey Fc Dual-Mode Imaging System (LI-COR Biosciences). Band density analysis was conducted using Image Studio Lite v. 5.2 software (LI-COR Biosciences) [18].

### 2.5. Determination of IκBα Levels via Flow Cytometry

bFLSs were cultured in 6-well plates and incubated in the presence or absence of 50 μM of andrographolide for 30 min. Afterwards, cells were stimulated with 5 mM of D-lactate for 45 min or 100 ng/mL of bTNF-α for 15 min at 37 °C in a 5% CO_2_ environment. Post-stimulation, cells were harvested through trypsinization and fixed in 4% paraformaldehyde for 15 min. Subsequently, the cells underwent PBS washing and permeabilization in ice-cold 90% methanol. To assess alterations in IκBα levels, the cells were incubated with an AlexaFluor 488-conjugated IκBα mouse mAb (#5743, Cell Signaling Technology) for 1 h in 0.5% BSA in PBS pH 7.4. Flow cytometry analysis was conducted using a FACS Canto II flow cytometer (Becton Dickinson, Franklin Lakes, NJ, USA) and analyzed with FlowJo 7.6 software (TreeStar Inc., Ashland, OR, USA) [18].

### 2.6. Determination of Cell Viability by CCK-8 Proliferation Kit

To assess cell viability, 10,000 cells/well were cultured in a 96-well plate at 37 °C in a humidified environment with 5% CO_2_. After 24 h, bFLSs were incubated with a vehicle (0.1% DMSO), or 5 μM or 50 μM of andrographolide for 30 min. Afterwards, cells were stimulated with 5 mM of D-lactate or 100 ng/mL of bTNF-α for 6 h. Finally, 5 µL of CCK-8 reagent (Cayman Chemicals, Ann Arbor, MI, USA) was added to each well and incubated for 2 h. OD was measured at 450 nm using a Varioskan Flash Reader (Thermo Fisher Scientific).

### 2.7. Statistical Analysis

All assays represent the mean ± SEM of at least four independent experiments. A one-way analysis of variance (ANOVA) followed by Fisher’s LSD multiple comparison test was employed, considering a significance level of 5%. In cases where assumptions of normality or homogeneity of variance were not met (as assessed by the Shapiro–Wilk or Brown–Forsythe test, respectively), we applied the Kruskal–Wallis ANOVA and Dunn’s multiple comparison test. GraphPad Prism v. 7.0 (GraphPad Software, La Jolla, CA, USA) was used for all statistical analyses, and significance was established at a *p*-value < 0.05.

## 3. Results

### 3.1. Andrographolide Reduces the IL-8, IL-6, and COX-2 Expression Induced by D-Lactate and TNFα in bFLSs

**Figure 1:**
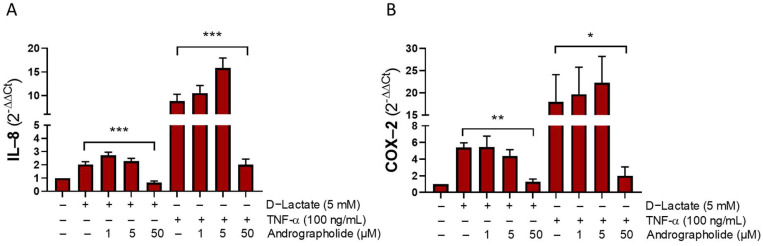
Andrographolide interferes with the expression of IL-8 and COX-2 induced by D-lactate and TNFα in bFLSs. Relative expression of IL-8 (**A**) and COX-2 (**B**) mRNA in bFLS cells treated with D-lactate or bovine TNFα for 6 h. Each bar represents the mean ± the standard error of media (SEM). *n* = 4, * *p* < 0.05; ** *p* < 0.01, *** *p* < 0.001.

We assessed if andrographolide can inhibit the pro-inflammatory effects of D-lactate in bFLS. In fact, 50 μM of andrographolide significantly reduced the IL-8 and COX-2 expressions induced by D-lactate. As a control, we used the TNF-α cytokine that induces inflammatory cytokine production in a human synoviocyte [31]. Similarly, andrographolide significantly decreased the IL-8 and COX-2 expression induced by TNF-α in bFLSs (Figure 1).

**Figure 2:**
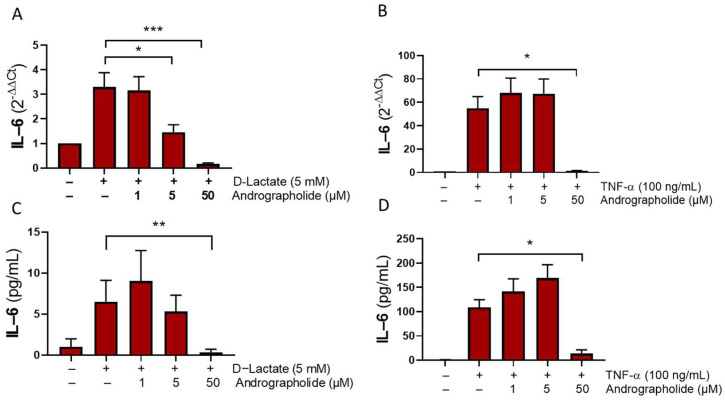
Andrographolide interferes with the expression and release of IL-6 induced by D-lactate and TNFα in bFLSs. Relative expression of IL-6 (**A**,**B**) and release of IL-6 (**C**,**D**) in bFLS treated with D-lactate or bovine TNFα for 6 h. Each bar represents the mean ± the standard error of media (SEM). *n* = 4, * *p* < 0.05; ** *p* <0.01; *** *p* < 0.001.

Lactate plays a pivotal role in regulating IL-6 secretion in fibroblast-like synoviocytes (FLSs), resulting in increased IL-6 levels in human synovial fibroblasts [15]. Our study demonstrates that andrographolide significantly inhibits the IL-6 elevation induced by D-lactate. Additionally, andrographolide reduced the secretion triggered by TNF-α in bFLS, suggesting that the anti-inflammatory effect is not exclusive to D-lactate (Figure 2).

Some previous studies suggest that andrographolide can induce apoptosis in RA-FLS [32]. To eliminate the possibility of any impact on cellular viability, we assessed whether andrographolide reduces the viability of bFLS. Andrographolide at 6 h did not induce cytotoxicity effects in bFLS (Figure S1), suggesting that the anti-inflammatory effects observed might be more reliant on the interference with the signal transduction induced by D-lactate and TNFα. We previously demonstrated that D-lactate increases IL-6 production in bFLS, activating PI3K/Akt and NF-κB pathways [18]. For this reason, we investigated whether andrographolide has the potential to disrupt the induction of IL-6 by D-lactate in bFLS.

### 3.2. Andrographolide Reduces the Akt Phosphorylation and Interferes with the IkBa Degradation Induced by D-Lactate and TNFα in bFLS

It has been demonstrated that D-lactate induces Akt phosphorylation in bFLS within 5 min of stimulation [18]. Additionally, lactate can induce the PI3K/Akt pathway in human chondrocytes [33].

**Figure 3:**
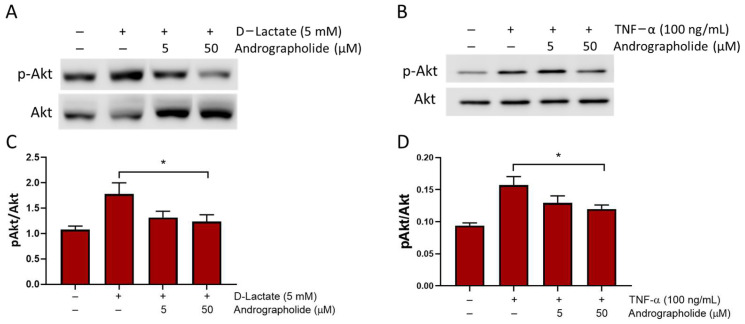
Andrographolide inhibits the D-lactate and TNFα-induced phosphorylation of Akt in bFLS. bFLSs were treated with 5 mM of D-lactate (**A**,**C**) and TNFα for 5 min (**B**,**D**). Total protein was analyzed by immunoblotting using specific antibodies against the phosphorylated forms of Akt (pAkt). Total Akts were also evaluated via Western blot for comparison. Images of a representative experiment are shown. The densitometry ratios of pAkt/Akt are shown in the graphs as mean ± standard error of mean (SEM) (*n* = 3), * *p* < 0.05.

Therefore, we assessed whether andrographolide could interfere with this signaling pathway in bFLSs. In our study, andrographolide diminished the Akt phosphorylation induced by 5 mM of D-lactate in bFLSs (Figure 3A). In addition, we observed that andrographolide significantly reduced the Akt phosphorylation induced by TNFα in bFLSs (Figure 3B).

**Figure 4:**
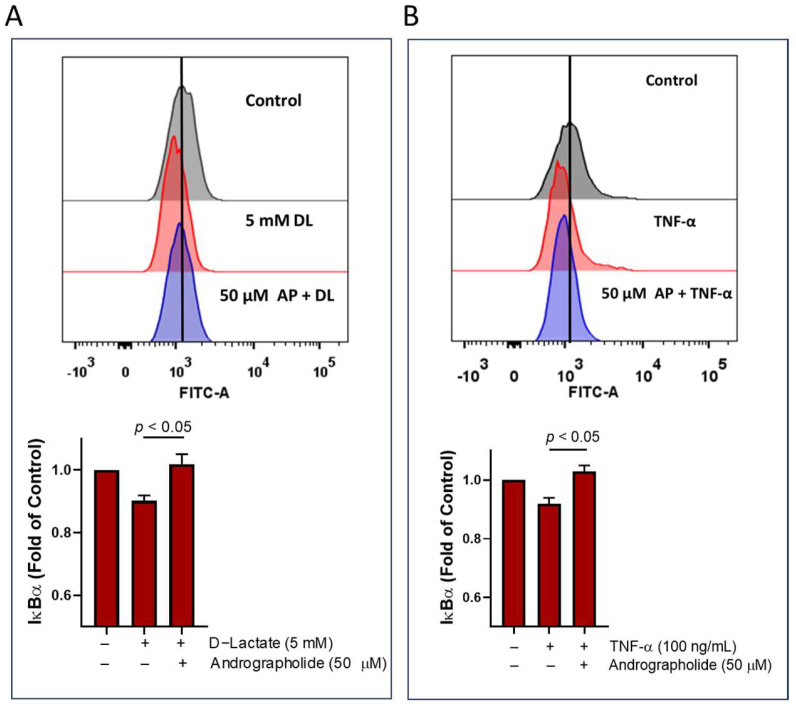
Andrographolide reduces the IκBα degradation induced by D-lactate and TNFα. IκBα degradation was evaluated in bFLS treated with (**A**) D-lactate (DL) and (**B**) bTNF-α by flow cytometry, in the presence or absence of 50 μM of andrographolide. Bar graphs represent the mean ± the standard error of mean (SEM) of four independent experiments.

The bovine IL-6 gene [34,35] contains promoter regions sensitive to NF-κB, primarily regulated upstream by the phosphatidylinositol 3-kinase (PI3K) pathway in synoviocytes [31]. Moreover, we previously demonstrated that D-lactate induces the expression of proinflammatory cytokine via the NF-κB pathway [18]. We conducted an assay to determine whether andrographolide can inhibit this pathway by measuring the degradation of IκBα using flow cytometry. We observed that 50 μM of andrographolide interfered with the degradation of IκBα induced by D-lactate and TNFα in bFLS (Figure 4).

### 3.3. Andrographolide Decreases the Expression of HIF-1 and LDHA Induced by D-Lactate

**Figure 5:**
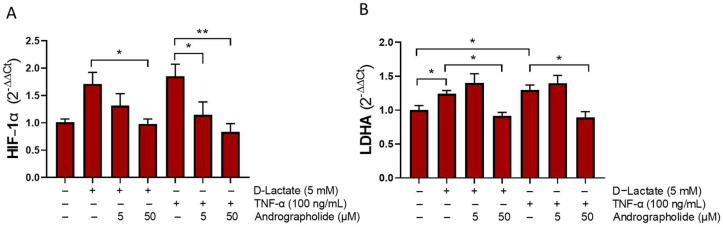
Andrographolide reduces the expression of HIF-1α and LDHA induced by D-lactate and TNFα. bFLS were preincubated with andrographolide and stimulated with D-lactate or TNFα for 6 h. The relative mRNA expression levels of HIF-1α (**A**) and LDHA (**B**) are shown. Each bar represents the mean ± the SEM, *n* = 5. * *p* < 0.05; ** *p* < 0.01.

In the bFLSs, treatment with 5 mM of D-lactate and TNFα significantly increased the expression of HIF-1α and LDHA. Interestingly, introducing 50 μM of andrographolide effectively reduced the expression of these genes (Figure 5). This finding suggests that andrographolide can partially reverse the metabolic reprogramming induced by these proinflammatory agents.

## 4. Discussion

In this study, we found that treatment with 50 μM of andrographolide significantly reduced IL-8 and COX-2 expressions, which were induced by 5 mM of D-lactate in bFLS. Interestingly, andrographolide also reduced IL-8 and COX-2 expressions when stimulated by TNFα. This finding suggests that andrographolide’s ability to suppress these inflammatory markers in bFLSs is not exclusive to D-lactate stimulation but also other pro-inflammatory stimuli. Andrographolide reduces the IL-8 expression induced by TNFα in HCT116 cells [36] and inhibits COX-2 expression in macrophages [37,38] and neutrophils [39].

In cattle, D-lactate production occurs via two primary pathways: the methylglyoxal metabolic pathway and digestive fermentative disturbances during ruminal acidosis [1,5,6,40]. The former involves the breakdown of glucose and sugars, generating methylglyoxal, which subsequently metabolizes into D-lactate [1,40]. Conversely, ruminal fermentative disorders lead to the excessive accumulation of D-lactate, disrupting normal metabolic processes and causing pathological effects such as polysynovitis [1,5]. The presence of lactate within the joint during arthritis progression is considered a pivotal indicator linked to the inflammatory cascade, currently emerging as a promising target for the advancement of novel therapeutic interventions [3,41,42]. Lactate induces the release of Neutrophil Extracellular Traps (NETs) [43] and activates the secretion of pro-inflammatory cytokines in FLS [15,17], thereby contributing to the inflammation observed in arthritis. Furthermore, D-lactate can trigger the cellular signaling that induces NETs [8,44,45] and the production of inflammatory mediators in bFLS [11,19,46], which are detected in the synovial fluid from heifers with acute ruminal acidosis (ARA) [12].

We observed that andrographolide could interfere with the IL-6 expression induced by D-lactate and TNFα stimulation. IL-6 triggers the immune system, promoting an inflammatory response. In joints, IL-6 expression shows a positive correlation with the severe lesions observed in aseptic joint conditions, such as rheumatoid arthritis or osteoarthritis [47,48,49]. In heifers with experimentally induced ruminal acidosis, a marked increase in IL-6 concentration was observed in the synovial fluid compared to the plasma. This elevation is significantly correlated with the presence of D-lactate specifically detected in the inflamed joints of these animals [11]. The inhibitory effect of andrographolide on IL-6 has been demonstrated in RAW264.7 cells, primary alveolar type II epithelial cells, and HepG2 cells [38,50,51]. This effect is closely attributed to its capability to reduce the activity of the PI3K and NF-κB pathways [38,50,51].

In the present study, we demonstrated that andrographolide reduces the PI3K/Akt and NF-κB pathway in bFLSs. Lactate can activate the PI3K/Akt pathway in the human chondrocyte through receptor HCA1 [33]. Moreover, when this pathway was inhibited by the use of LY294002, a PI3K inhibitor, a reduction in the IL-6 and IL-8 production induced by D-lactate was observed, suggesting that the PI3K/Akt pathway is critical for the pro-inflammatory effects of D-lactate in bFLS [18]. Furthermore, andrographolide reduces the TNFα-induced Akt phosphorylation in HUVEC cells, decreasing the intercellular adhesion molecule-1 (ICAM-1) expression that is key to neutrophil migration [52]. Conversely, D-lactate increases neutrophil adhesion to the vascular endothelium via CD11b/ICAM-1 [44]. Since ARA has been shown to increase neutrophils in heifers’ synovial fluid [10,12], andrographolide’s inhibition of PI3K and its effects on various biological processes associated with this pathway and inflammation may be beneficial in controlling polysynovitis in cattle affected by ARA.

Andrographolide reduced the degradation of the IκBα induced by D-lactate and TNF-α in bFLS. We previously demonstrated that BAY 11-7082, which irreversibly inhibits IKKα and reduces the phosphorylation and degradation of IκBα, reduces D-lactate and TNF-α-induced IL-6 overexpression in bFLSs [18]. Andrographolide exerts an anti-inflammatory effect by reducing the degradation of the IκBα induced by TNFα in THP-1 cells [24]. Upon interaction with TNFα receptor 1 (TNFR1) receptors, various adaptors initiate the activation of the IκB-kinase (IKK) complex [53]. Subsequently, the activated IKK phosphorylates the IκBα. Following phosphorylation, IκBα undergoes ubiquitination and degradation facilitated by the 26S proteasome. The NF-κB dimer (p65/p50), once liberated from IκBα, can bind to DNA sites located on the IL-6 gene promoter [35]. Moreover, the NF-κB pathway activation plays a crucial role in maintaining the constant expression levels of IL-6 and IL-8. It also orchestrates the secretion of these cytokines, stimulated by IL-1β, in RA-FLSs [54].

Andrographolide reduced the expression of HIF-1α and LDHA induced by D-lactate and TNF-α in bFLS. This suggests its potential role in modulating the metabolic changes triggered during joint inflammation. In osteoarthritis and rheumatoid arthritis, human FLS demonstrates an altered glycolytic metabolism, contributing to increased lactate production [55,56]. Lactate can serve as a signaling molecule capable of triggering inflammation during arthritis [17] and inducing metabolic rewiring in FLS [33]. In connection with this, lactate in human RA-FLS [18,19] and D-lactate in bFLS [1,15] increase glycolysis and IL-6 production. Compared to normal subjects, RA patients exhibit an increase in metabolic enzymes pivotal to glycolysis, including LDHA. This elevation has been observed to correlate with higher levels of lactate in the synovial fluid [57]. A metabolomic analysis conducted via gas chromatography-mass spectrometry (GC-MS) unveiled disruptions in the metabolic pathways of bFLSs induced by D-lactate. Notably, D-lactate primarily enhances glycolysis, gluconeogenesis, the pyruvate metabolism, and the galactose metabolism in these cells [19]. The RT-qPCR analysis demonstrated elevated expression levels of metabolic-related genes, HIF-1α, and LDHA [19]. TNF-α also induces metabolic reprograming increasing glycolysis, the expression of HIF-1 and IL-6 [58]. Recently, we demonstrated that TNFα also induces an increase in glycolysis and in HIF-1α and LDHA expressions in bFLSs [46]. Moreover, FX11, an LDHA inhibitor, reduced IL-6 expression [46]. These findings suggest that metabolic reprogramming plays a pivotal role in the proinflammatory effect observed in bFLS induced by both D-lactate and TNFα. In addition, the intra-articular administration of oxamate, acting as an LDHA inhibitor, in a murine osteoarthritis model demonstrated reduced pain and inflammation, alongside an improvement in metabolic reprogramming [59]. Moreover, it has been demonstrated that knocking down HIF-1α in RA-FLS led to a reduction in LDHA and phosphofructokinase expression [57]. Thus, andrographolide could exert its anti-inflammatory effects by modifying the metabolism during inflammatory processes. This finding is further supported by the observation that pyruvate kinase M2 (PKM2) function, a glycolytic enzyme responsible for converting phosphoenolpyruvate (PEP) to pyruvate, is elevated in RA synovial tissues and osteoarthritis chondrocytes, a promising new avenue for therapeutic targeting [60,61]. Initial observations within the synovial fluid during ARA induction via oligofructose overload reveal a notable surge in pyruvate, lactate, and IL-6 concentrations [11]. These findings indicate that an elevation in glycolysis may represent an early stage of synovitis development in cattle. Studies with quantitative chemical proteomic approaches have also unveiled novel potential targets for andrographolide within the realms of inflammation and immunity, including PKM2 and LDH [62]. Other authors suggest that andrographolide contributes, at least partially, to preserving gastric vascular homeostasis by inhibiting the PFKFB3-mediated glycolysis pathway during gastric ulcer development [63].

The inhibition of the PI3K/Akt, NF-κB, and HIF-1α pathways may be intricately linked to the potential restoration of glycolytic reprogramming in FLSs [46,64]. Addressing both the inflammatory cascade and metabolic changes during arthritis represents an emerging scientific approach to alleviate joint inflammation [41,42]. Andrographolide, by modulating diverse cellular pathways such as PI3K/Akt, NF-κB, and HIF-1 [29,65,66], could exhibit an enhanced anti-inflammatory efficacy due to its multitarget effects on cellular metabolism [67]. Collectively, these findings suggest that andrographolide may mitigate joint inflammation, thereby reducing the metabolic alterations observed during ARA [11], and thus emerging as a promising candidate for addressing inflammatory processes in cattle joints.

## 5. Conclusions

Our results highlight the role of andrographolide as a potential therapeutic agent for modulating the metabolic alterations associated with inflammation. By targeting the PI3K/Akt, NF-κB, and HIF-1α pathways, andrographolide reduced the expression of key genes involved in inflammation (IL-6, IL-8, and COX-2) and the glycolytic metabolism, such as LDHA. Therefore, andrographolide can help restore metabolic homeostasis in bFLSs and attenuates the inflammatory processes associated with joint diseases during ARA.

## Data Availability

The data presented in this study are available on request from the corresponding author.

## Supplementary Materials

The following supporting information can be downloaded at: https://www.mdpi.com/article/10.3390/ani14060936/s1, Figure S1: Effect of andrographolide on bFLS viability.
