# Supplementary material for: Andrographolide Ameliorates Inflammatory Changes Induced by D-Lactate in Bovine Fibroblast-like Synoviocytes
Source: Animals (Basel). 2024 Mar 19;14(6):936. doi: 10.3390/ani14060936 (PMC10967535; doi:10.3390/ani14060936)
Supplement: Supplementary file 1 [file animals-14-00936-s001.zip › animals-2802167-supplementary.pdf]

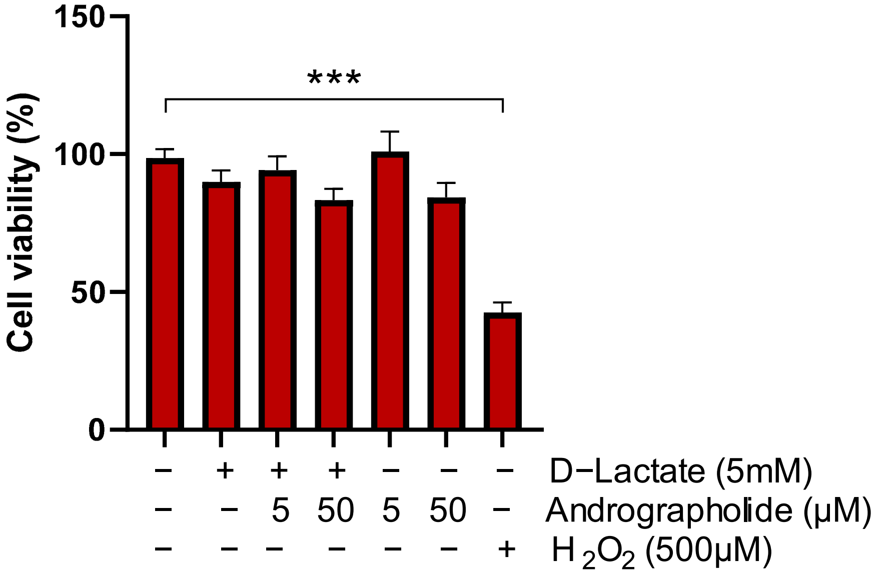

Figure S1: Effect of andrographolide on bFLS viability. bFLSs were preincubated with andrographolide or vehicle (0.1% DMSO) for 30 min. and then stimulated with D-lactate or TNFα for 6 h. Cellular viability was measured using CCK-8 kit. H_2O_2 was used as control for cytotoxicity. Each bar represents the mean ± SEM, n = 5. ***$p < 0.001$
